# Giant Prostate Utricle Cyst

**DOI:** 10.5334/jbsr.1738

**Published:** 2019-02-05

**Authors:** Bert Degrieck, Chloë Standaert, Geert Villeirs

**Affiliations:** 1 UZ Gent, BE

**Keywords:** prostate utricle cyst, Müllerian duct remnant, prostate, prostate cyst, prostatitis

## Case Report

**Figure 1:**
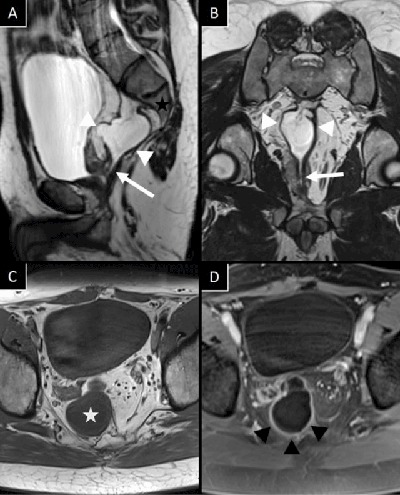
Sagittal **(A)** and paracoronal reformatted **(B)** T2-weighted images show a large thick-walled presacral cystic mass (arrowheads) with a thin anteroinferior neck (arrows) connecting to the prostatic urethra at the level of the verumontanum. The cystic component has a homogeneous high T2 signal intensity, with a low T2 signal intensity cyst wall. Axial T1-weighted image **(C)** shows low signal intensity of the cyst (white asterisk), similar to the bladder content, excluding hemorrhagic/proteinaceous components. Axial fat saturated T1-weighted vibe image after intravenous contrast administration **(D)** shows only contrast enhancement of the cyst walls (black arrowheads). There is a right-sided displacement of the prostate secondary to surgical pull-through intervention as treatment for the anal atresia. Also note the congenital sacral deformity (black asterisk on A).

A 30-year-old male with history of repaired hypospadias and anal atresia was referred for magnetic resonance imaging (MRI) of the pelvis (consisting of T1-weighted, T2-weighted and dynamic contrast-enhanced [DCE] images) because of ongoing chronic prostatitis-like complaints for three months. MRI revealed a large thick-walled cystic lesion in the midline between the prostate and the (deformed) sacrum, communicating with the prostatic urethra at the level of the verumontanum via a thin neck (Figure 1). Neither T2w nor DCE images showed signs of prostatitis. The diagnosis of a giant utricle cyst with chronic superinfection was proposed. Urethroscopy confirmed the connection between the cystic lesion and the verumontanum. The aspirated fluid was turbid, suggesting chronic infection. Robot-assisted surgical marsupialization of the cyst was performed, with deroofing of the cyst wall as well as closure of the communication between the neck of the cyst and the verumontanum. The procedure was complicated with pelvic abscess formation and perforation of the bladder wall in the following days. The complications were managed conservatively, and control cystography performed one month later showed closure of the bladder defect.

## Discussion

Prostate utricle cysts (PUCs) are cystic intraprostatic lesions located dorsocranially in the midline with communication to the prostatic urethra at the level of the verumontanum. They are embryologic remnants of the Müllerian duct system and are associated with genito-urinary abnormalities including hypospadias, intersex disorders, cryptorchidism and renal agenesis [1]. PUCs are usually small (<10 mm), asymptomatic and pear-shaped, often seen as an incidental finding on prostate/pelvic imaging. Small PUCs are fairly common with an incidence of up to 5% of the general male population and are most commonly found in young males. PUCs do not normally extend beyond the base of the prostate. Large PUCs are very rare and can present with postvoiding incontinence, (recurrent) urinary tract infection, hematospermia, (recurrent) epididymitis or pain. When infected, they can contain pus or hemorrhage, making it hard to differentiate them from abscess or cystic tumor on imaging studies. Differential diagnosis includes a seminal vesicle cyst or bladder diverticulum. Treatment of symptomatic PUCs include cyst aspiration (transperineal or transrectal), endoscopic resection, surgical resection or cyst marsupialization.
